# Can Neutrophil-Lymphocyte Ratio and Lymph Node Density Be Used as Prognostic Factors in Patients Undergoing Radical Cystectomy?

**DOI:** 10.1155/2013/703579

**Published:** 2013-03-31

**Authors:** Abdullah Demirtaş, Volkan Sabur, Emre Can Akınsal, Deniz Demirci, Oguz Ekmekcioglu, Ibrahim Gulmez, Atila Tatlisen

**Affiliations:** Department of Urology, Erciyes University Medical Faculty, 38039 Kayseri, Turkey

## Abstract

*Objective*. To assessment the role of preoperative neutrophil-lymphocyte ratio and postoperative lymph node density in predicting prognosis in patients undergoing radical cystectomy for bladder cancer. *Material and Methods*. Preoperatively, neutrophil and lymphocyte counts as well as neutrophil-lymphocyte ratios were recorded in 201 patients who underwent radical cystectomy for bladder cancer. Patients with an infection were excluded. Based on the pathology reports, the number of positive lymph nodes was divided by the total number of lymph nodes to calculate lymph node density. *Results*. The mean follow-up duration was 37.22 ± 35.922 months in patients without lymph node involvement and 27.75 ± 31.501 months in those with lymph node involvement (*P* = 0.015). Median lymph node density was 17% (4–80) in patients with lymph node involvement. There was no difference according to lymph node density lower than 17% and greater than 17% (*P* = 0.336). There was no significant difference between patients with an NLR below or above 2.5 in terms of overall survival (*P* = 0.702). Pathological T stage was associated with survival (*P* = 0.004). *Conclusion*. In patients undergoing RC for bladder cancer, lymph node density and preoperative NLR were not found to be independent predictors of prognosis.

## 1. Introduction 

According to the European survey for cancer incidence and mortality in 2006 the incidence of bladder cancer among all cancers is 6.6% in men and 2.1% in women. Cancer-related mortality is 4.1% in men and 1.8% in women [1]. In cancers with muscular invasion the gold standard treatment method is radical cystectomy (RC). In addition, radical cystectomy may also be performed in patients with a cancer at stage Ta-1 or Tis that is resistant to chemotherapy and/or BCG treatment. Lymph node (LN) dissection is an integral part of radical cystectomy. It is beneficial for staging and survival [2]. 

Following studies by Herr [3] and Stein et al. [4] LN density has been related to prognosis. The ratio of the number of positive LNs to the total number of excised LNs is defined as LN density.

Systemic inflammation is characterized by fever, leucocytosis, and elevated levels of serum acute phase proteins [5]. A more general and less specific immune response may be elicited due to tissue injury and distortion created by the physical effects of the tumor [6]. Neutrophil-lymphocyte ratio (NLR) has been suggested as a simple marker of systemic inflammatory response in critical care patients [7]. A higher NLR in the preoperative period has been reported as a useful parameter associated with poor prognosis in some cancers including bladder cancer [8–10].

In this study we aimed to determine whether NLR, a marker of inflammatory response, and lymph node density calculated after surgery are prognostic factors in patients with bladder cancer undergoing radical cystectomy. 

## 2. Materials and Methods

The data of 263 patients who underwent radical cystectomy for bladder cancer at our clinic between January 1999 and January 2013 were retrospectively analyzed. The data of 201 patients matching the inclusion criteria were assessed. In addition to patients undergoing transurethral resection for bladder tumor (TUR-BT) and with a pathology consistent with T2, patients with pathology consistent with Ta-1 or Tis who were resistant to intracavitary chemotherapy and/or the Bacillus Calmette-Guérin (BCG) therapy were also operated on if an indication existed according to contemporary guidelines (CTa-4NxM0). Patients with an infection, a second primary cancer, a bladder cancer other than the urothelial cancer subtype, a hematologic disorder with the potential to alter the neutrophil-lymphocyte ratio, and missing data were excluded. Preoperative hemoglobin (Hb), neutrophil count, lymphocyte count as well as postoperative total number of excised lymph nodes, the number of positive lymph nodes, follow-up durations, status of adjuvant therapies, and death rates were recorded. For each patient a neutrophil-lymphocyte ratio was calculated [11]. Lymph node density level was calculated for those with lymph node involvement. Lymph node density was calculated by dividing the number of positive lymph nodes by the number of total lymph nodes [3, 4].

Normality of the study data was tested with the Shapiro-Wilk test. The relationship between the variables was tested using the Spearman test. Survival analyses were performed with the Kaplan-Meier analysis, and risk factors were determined with Cox regression analysis. A *P* value lower than 0.05 was considered statistically significant.

## 3. Results

**Figure 1:**
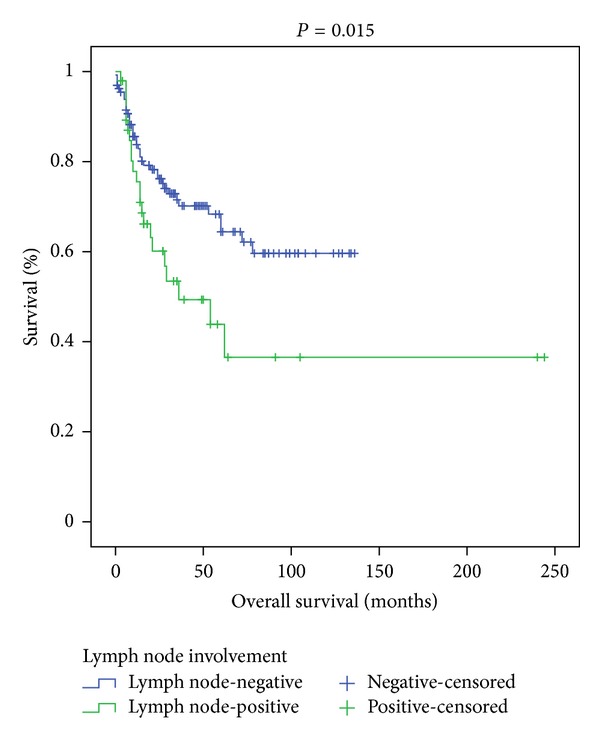
The Kaplan-Meier curves according to lymph node involvement (*P* = 0.015).

**Figure 2:**
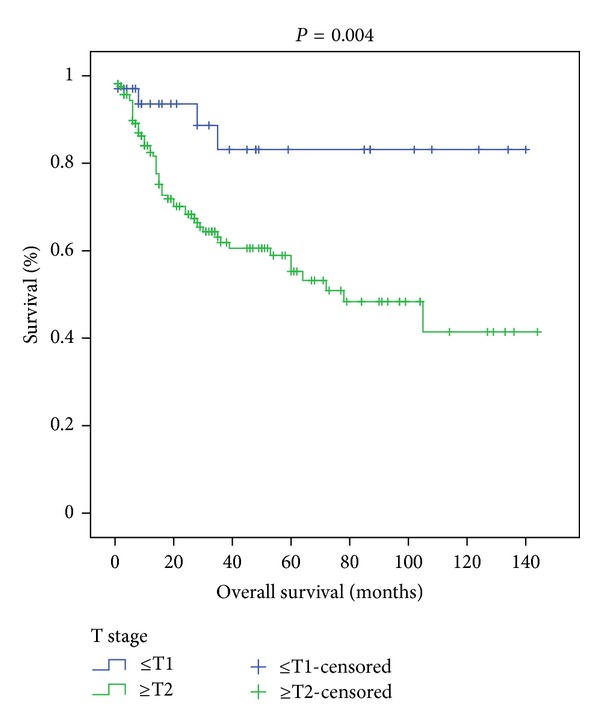
The Kaplan-Meier curves according to T stage (*P* = 0.004).

Among the 201 patients, 175 were male, and 26 were female. The mean age of the patients was 62.03 ± 9.42 years (34–82 years). During the radical cystectomy operation an orthotopic bladder (sigmoid neobladder) was created in 56 patients, an ileal loop in 5 patients, and ureterocutaneostomy in 140 patients. Only 2 patients were given neoadjuvant chemotherapy. The pathology reports of the radical cystectomy specimens were consistent with lymph node involvement in 55 patients. Thirty-eight patients in the LN-negative group and 23 patients in the LN-positive group died. Twenty-seven of the LN-positive patients were given chemotherapy, 3 patients were given radiotherapy, and 4 patients were given chemoradiotherapy adjuvant therapies. Some of the other patients were not considered suitable for additional therapy owing to their general condition, and others did not give consent for additional therapy. Among the LN-negative group, 11 patients were given chemotherapy, 4 patients were given radiotherapy, and 3 patients were given chemoradiotherapy as adjuvant therapy. The characteristic features of the patients are given in Table 1. The mean duration of followup was 37.22 ± 35.922 months in LN-negative patients and 27.75 ± 31.501 months in LN-positive patients (*P* = 0.015) (Figure 1). The patients were classified into 2 groups according to T stage. The survival rates of 35 patients in the T0, Ta, and T1 groups and 166 patients in the T2, T3, and T4 groups were assessed with the Cox regression and Kaplan-Meier analyses. The first group had a significantly longer survival (*P* = 0.004) (Figure 2). Both groups were also compared with each other in terms of NLR. There was no statistically significant difference between the two groups in terms of NLR (*P* = 0.533) (Table 2).

The mean number of excised LNs was 13.39, while the mean number of excised LNs among those with lymph node involvement was 16.55. Variables in terms of lymph node involvement are given in Table 3. The median level of lymph node density was 17% (4–80). The mean follow-up duration was 38.8 months in 28 patients with a lymph node density below 17% and 31.3 months in 27 patients with a lymph node density above 17% (*P* = 0.336). 

The preoperative peripheral hemoglobin level of the patients averaged 12.57 ± 2.15 gr/dL, neutrophil count was 6095 ± 2446 *µ*L, lymphocyte count was 1764 ± 781 *µ*L, and NLR was 4.43 ± 4.11. Pretreatment NLR was found to be significantly correlated with Hb level (*P* < 0.001), neutrophil count (*P* < 0.001), and lymphocyte count (*P* < 0.001). There was also a significant correlation between Hb level and lymphocyte count (*P* < 0.001). Age was not significantly correlated with overall survival (*P* = 0.388). Among the entire study population, there was no significant difference between those with NLR above or below 2.5 in terms of overall survival (*P* = 0.702). No significant difference existed between patients with or without lymph node involvement when NLR was above versus below 2.5 (*P* = 0.420). Patients with a lymph node density equal to or lower than 17% were not different from those with a lymph node density above 17% in terms of NLR (*P* = 0.4) (Table 2). The one-, three-, and five-year survival rates were 84.4%, 70.7% and 64.9% in the lymph node-negative patients, respectively. On the other hand, the one-, three-, and five-year survival rates were 88.3%, 48.9%, and 36.3% in the lymph node-positive patients, respectively.

## 4. Discussion

Urinary bladder cancer is the ninth most common cancer worldwide. Each year 330,000 new cases are diagnosed and 130,000 patients die from this disease [12, 13]. Seventy percent of cases do not exhibit muscle invasion at diagnosis, whereas 30% had muscle invasion at the time of diagnosis. Approximately 43% of patients undergoing radical cystectomy for muscle-invasive bladder cancer underwent radical cystectomy after the cancer became muscle-invasive but limited to that organ while the patients were attending followup for superficial bladder cancer. The remaining 57% were diagnosed with muscle-invasive bladder cancer at the time of initial diagnosis and therefore proceeded to radical cystectomy [13].

Recently, many prognostic factors have been defined in bladder cancer. Most efforts have been directed at prediction of survival after radical cystectomy with the help of postoperative parameters such as pT, pN, and lymphovascular invasion [10]. Some studies have demonstrated that lymph node involvement unfavorably affects survival. In 2006 Hautmann et al. studied 788 patients undergoing radical cystectomy and found that the 5- and 10-year recurrence-free survival rates in patients without lymph node involvement were 74.8 and 67.9%, respectively. The same figures in 142 patients with lymph node involvement were 20.9% and 14.6%, respectively (*P* < 0.0001) [14].

Madersbacher et al. (2003) and Dalbagni et al. (2001) reported that lymph node involvement following radical cystectomy affects survival unfavorably [15, 16]. Another study detected survival rates of 38.7% and 78.3% at three years in patients with, versus without, LN involvement, respectively [17]. We also found a significant difference between those with, versus without, lymph node involvement in terms of overall survival (*P* = 0.015).

There is no consensus in the literature as to the number and regions of LN excision. In one study it was found that survival was favorably affected by the excision of more than 10 LNs in LN-positive patients [18]. In our study, the average number of excised LNs was 13.39, and this was consistent with the literature. 

Herr and Stein, in their 2003 work, used the term “lymph node density” for the first time and reported that the excision of a high number of lymph nodes had a favorable effect on survival, even when lymph node involvement was present. Herr found a 5-year survival rate of 64% in patients with a lymph node density equal to or lower than 20% and 8% in those with a lymph node density greater than 20% (*P* < 0.001) [3]. Another study by Stein et al. in the same year accepted a cut-off level of 20% for lymph node density. The 10-year overall survival was 43% in patients with an LN density equal to or lower than 20% whereas it was 17% for those with an LN density greater than 20% (*P* < 0.001) [4]. Leissner et al. reported a decreased local recurrence rate with increasing number of LN despite the presence of lymph node involvement [19]. We assumed a cut-off level of 17% in our study since the median value for lymph node density equalled 17%. We detected no statistically significant difference between the patient group with an LN density lower than 17% and the group with a lymph node density greater than 17% in terms of survival (*P* = 0.336). There was no a significant difference detected when the cut-off level was assumed as 20% (*P* = 0.772). The reason for the lack of relationship between LN density and survival in our study may be explained by the small sample size. In addition, it may also depend on whether a standard, versus extended, lymph node dissection was carried out. Prospective studies of standardized lymph node dissection should be done to ascertain the prognostic value of LN density.

Although there are numerous studies aimed at the prediction of survival after radical cystectomy in bladder cancer, studies aiming to predict survival rates before radical cystectomy are rather retrospective, scarce, and inadequate. Karakiewicz et al. developed a preoperative nomogram in 2006 to predict pT and pN, although that study did not aim to predict survival [20], and it could not completely fill the scientific gap in this field. In 2012 Gondo et al. demonstrated that the presence of hydronephrosis and simultaneous carcinoma in situ as well as tumor size were independent predictors for survival. They also reported that, in addition to those clinical and pathological criteria, laboratory data such as CRP, lymphocyte and neutrophil counts, hemoglobin level, and NLR were also associated with survival. They found that, on multivariate analysis, NLR and hemoglobin level were independent predictors for survival [10]. Previous studies have shown that a high NLR is a poor prognostic sign in malignancies of the stomach [21], liver [22], and kidney [9], small cell lung cancer [23], and ovarian cancer [24]. Can et al. conducted a study to test whether NLR was a predictor for the invasiveness of bladder cancer. In that study, 182 patients with bladder cancer were included and assessed retrospectively. Pathological examination of those cases revealed that the cancers in 80 patients were not muscle-invasive whereas those in 102 patients were. They found that such factors as tumor size, number of tumors, occupational status, and life style failed in predicting the invasiveness of bladder cancer whereas NLR, thrombocyte count, age, and gender were significantly related with the prediction of pathological stage of the tumor [25]. In our study preoperative NLR was significantly correlated with Hb level (*P* < 0.001) and neutrophil (*P* < 0.001) and lymphocyte (*P* < 0.001) counts. In addition, there was a significant correlation between Hb level and lymphocyte count (*P* < 0.001). However, no significant relationship could be found between survival and age (*P* = 0.7), hemoglobin level (*P* = 0.207), and neutrophil (*P* = 0.805) and lymphocyte (*P* = 0.311) counts. Moreover, our study concluded that NLR was not a powerful predictor of survival in patient groups with versus without lymph node involvement (*P* = 0.420), in patient groups with a lymph node density lower versus greater than 17% (*P* = 0.4), or in patient groups with a pathological stage ≤T1 versus ≥T2 (*P* = 0.533). In the study of Gondo et al. many clinical factors were related with survival although clinical T stage fell short in predicting survival in a multivariate analysis. In the same study pathological T stage was not included in the study [10]. We did, on the other hand, detect that pathological T stage was a significant predictor of survival (*P* = 0.004).

## 5. Conclusion

Evidence from our study, as well as other studies, suggests that T stage and lymph node involvement appear as important factors in predicting survival in patients undergoing radical cystectomy. In this study, preoperative hemoglobin, neutrophil and lymphocyte counts, NLR, and postoperative LN density were not related with prognosis. The limitations of our study include its retrospective nature, limited sample size, and lack of assessment of parameters such as CRP, presence of hydronephrosis, size of the primary tumor, and number of foci. More prospective studies with a larger sample size are needed on this subject. 

## Figures and Tables

**Table 1:** Characteristic features of patients.

Parameters	*n* = 201
Mean age (years)	62.03 ± 9.42 (34–82)
Gender	
Male	175
Female	26
T stage	
≤pT	35
≥pT	166
N stage	
pN1	27
pN2	24
pN3	4
Chemotherapy	38
Radiotherapy	7
Chemoradiotherapy	7
No. of LN removed	13.39 (1–44)
No. of positive LN	3.61 (1–20)
LN density (%)	17 (4–80)
	61

**Table 2:** NLR and overall survival rate according to T stage and LN density.

	T stage			LN density		
	≤T1	≥T2	*P* value	≤17%	>17%	*P* value
NLR	4.5247 ± 4.4280	4.4192 ± 4.0625	0.533	3.07 (0.45–8.80)	3.16 (1.50–37.70)	0.400
Overall survival (months)	39.34 (2–134)	33.21 (1–144)	0.004	38.8 (3–105)	31.3 (3–144)	0.336

**Table 3:** Variables according to LN involvement.

Variables	LN involvement		*P* value
	Positive (+)	Negative (−)
Age (years)	62.64 ± 10.087	61.79 ± 9.183	0.147
Follow-up period (months)	27.75 ± 31.501	37.22 ± 35.922	0.015
Hemoglobin (gr/dL)	12.87 ± 2.354	12.45 ± 2.069	0.939
Neutrophil (*µ*L)	5.420 (4.310–6.890)	5.345 (4.502–7.200)	0.995
Lymphocyte (*µ*L)	1.720 (1.330–2.140)	1.600 (1.277–2.167)	0.497
NLR	3.08 (2.3498–4.519)	3.60 (2.245–5.180)	0.569

*Values are expressed as mean ± SD or median (25th–75th percentiles).
